# Early diagnostic value of survivin and its alternative splice variants in breast cancer

**DOI:** 10.1186/1471-2407-14-176

**Published:** 2014-03-12

**Authors:** Salma Khan, Heather Ferguson Bennit, David Turay, Mia Perez, Saied Mirshahidi, Yuan Yuan, Nathan R Wall

**Affiliations:** 1 Department of Biochemistry, Loma Linda University School of Medicine, Loma Linda, CA, USA; 2 Center for Health Disparities & Molecular Medicine, Loma Linda University School of Medicine, 11085 Campus Street, Mortensen Hall Room 160, Loma Linda, CA 92350, USA; 3 Department of Pathology & Laboratory Medicine, Loma Linda University School of Medicine, Loma Linda, CA 92350, USA; 4 Department of Medicine and LLU Cancer Center & San Manuel Band of Mission Indians Biospecimen Laboratory, Loma Linda University School of Medicine, Loma Linda, CA 92350, USA; 5 Division of Medical Oncology & Therapeutics, City of Hope Medical Center, Duarte, CA 91010, USA

**Keywords:** Survivin, Splice variants, Exosomes, Breast cancer

## Abstract

**Background:**

The inhibitor of apoptosis (IAP) protein Survivin and its splice variants are differentially expressed in breast cancer tissues. Our previous work showed Survivin is released from tumor cells via small membrane-bound vesicles called exosomes. We, therefore, hypothesize that analysis of serum exosomal Survivin and its splice variants may provide a novel biomarker for early diagnosis of breast cancer.

**Methods:**

We collected sera from forty breast cancer patients and ten control patients who were disease free for 5 years after treatment. In addition, twenty-three paired breast cancer tumor tissues from those same 40 patients were analyzed for splice variants. Serum levels of Survivin were analyzed using ELISA and exosomes were isolated from this serum using the commercially available ExoQuick kit, with subsequent Western blots and immunohistochemistry performed.

**Results:**

Survivin levels were significantly higher in all the breast cancer samples compared to controls (p < 0.05) with exosome amounts significantly higher in cancer patient sera compared to controls (p < 0.01). While Survivin and Survivin-∆Ex3 splice variant expression and localization was identical in serum exosomes, differential expression of Survivin-2B protein existed in the exosomes. Similarly, Survivin and Survivin-∆Ex3 proteins were the predominant forms detected in all of the breast cancer tissues evaluated in this study, whereas a more variable expression of Survivin-2B level was found at different cancer stages.

**Conclusion:**

In this study we show for the first time that like Survivin, the Survivin splice variants are also exosomally packaged in the breast cancer patients’ sera, mimicking the survivin splice variant pattern that we also report in breast cancer tissues. Differential expression of exosomal-Survivin, particularly Survivin-2B, may serve as a diagnostic and/or prognostic marker, a “liquid biopsy” if you will, in early breast cancer patients. Furthermore, a more thorough understanding of the role of this prominent antiapoptotic pathway could lead to the development of potential therapeutics for breast cancer patients.

## Background

Breast cancer is the most common cancer among women in the United States, and is the main cause of death in women 45 to 55 years of age with an estimated 226,870 new cases of invasive breast cancer expected to occur among women in the US this year [1,2]. The ultimate outcome of breast cancer relies on its initial stage at diagnosis with the main prognostic factors associated with breast cancer being lymph node involvement, tumor size and histological grade [3]. However, tumor at the same stage can behave in a different manner, and the prognosis can vary [4]. Therefore, it is important to find biomarkers that will predict the likelihood of recurrence and identify those patients who might benefit from additional therapy. Hence, low-risk patients can be spared unnecessary and costly treatment. Moreover, high-risk patients could be rapidly identified and offered appropriately aggressive treatment.

Survivin has been implicated in apoptosis inhibition and the regulation of mitosis in cancer cells. Survivin is overexpressed in a wide spectrum of tumors including prostate, pancreas, lung, ovarian, and breast cancer [5,6]. Recently, survivin expression in human breast cancers has been found using RT-PCR [7,8]. Like many genes, survivin is alternatively spliced with a number of new splice variants. Among them, survivin-2B is characterized by introducing a new exon of 69 bp with proapoptotic activity. Survivin-∆Ex3 has exon 3 excluded, and like the wild type, carries antiapoptotic activity. Survivin-3B has inclusion of a part of intron 3, preserving a complete BIR domain with antiapoptotic activity. Survivin-2α, the smallest survivin transcript, includes a 197 bp region of the 3′ end of intron 2, resulting in a truncated version of the BIR domain also having proapoptotic function [9,10]. Survivin and its splice variants are found to be associated with aggressive phenotypes of cancers [11] and may, if not prognostic, one day be considered like Her2 and hormone receptor to be predictive variables of breast cancer [3].

Survivin was recently shown to be released from cancer cells to the tumor microenvironment via small membrane bound vesicles (50-100 nm) called exosomes [12]. There has also been demonstrated a strong association of exosomal Survivin with high-risk prostate cancers (Gleason 6 and 9) as well as in cases becoming resistant to chemotherapy [13]. Although Survivin and its splice variants were shown differentially expressed in breast cancer tissues and were correlated with aggressiveness [6], their exosomal existence has not yet been addressed. Tumor-derived exosomes play multiple roles in tumor growth and metastasis and may produce their functions by influencing immune escape, tissue invasion and angiogenesis [14]. We have therefore undertaken the analysis of exosomal Survivin and its splice variants in breast cancer patient sera in parallel with paired breast tumor tissue. We believe exosomal investigation may provide novel biomarkers for early diagnosis of breast cancer when added to current recommended methods. Although Survivin and Survivin-∆Ex3 were detected in all of the samples examined, Survivin-2B was differentially expressed depending on the disease aggressiveness. Exosomal Survivin-2B (proapoptotic) was expressed mostly in primary tumors in early stage disease, whereas low or no expression was found in high-grade tumors. Survivin-2B was absent in most distant metastasis. We therefore believe that exosomal Survivin-2B can be further investigated as an early diagnostic or prognostic marker in breast cancer.

## Methods

### Patient serum

Serum samples were collected from forty breast cancer patients, ages ranging from 34-85 (median age 54) with clinical staging I-IV. Ten control samples from females who had undergone neoadjuvant treatment with either Zometa, Anastrazole, Taxol, Gemcitabine, Carboplatin and/or TCH followed by surgery with no recurrence were selected as controls. From the original 40 samples, 23 paired tissue and serum samples were obtained for splice variant analysis. All samples were collected through the San Manuel Band of Mission Indians Biospecimen Laboratory and the Cancer Center at Loma Linda University. Blood was collected in vacuum tubes containing sodium heparin. The tubes were centrifuged at 2000 × g for 7 minutes, and the serum was then removed and aliquoted for storage at -80°C.

### Patient consent

Written informed consent was sought and obtained from all participants involved in the course of these Loma Linda University Adventist Health Sciences IRB-approved studies in accordance with Loma Linda University policy.

### Human survivin immunoassay

Serum sample Survivin concentrations were quantitated using a commercially available human Survivin Immunoassay kit (R&D systems, Minneapolis, MN) according to the manufacturer’s instructions.

### Exosome isolation

For serum samples, the commercially available ExoQuick kit (SBI, Mountain View, CA) was employed. In brief, 100 μl of serum was incubated with 100 μl of ExoQuick solution followed by 2 hours incubation at 4°C followed by centrifugation at 1500 × g for 30 minutes. After centrifugation, the exosomes appear as a white pellet at the bottom of the vessel, which was then reconstituted with 500 μl of dH_2_O (Sigma, St. Louis, MO).

### Exosome quantification

To quantify the amount of exosomes released, we assessed the activity of acetylcholinesterase, an enzyme that is associated with these vesicles. Acetylcholinesterase activity was performed as described by Savina et al [15]. Briefly, 40 μl of the exosome fraction was suspended in 110 μl of PBS. 37.5 μl of this PBS-diluted exosome fraction was added to individual wells on a 96-well flat-bottomed microplate. Acetylthiocholine (1.25 mM) and 0.1 mM 5,50-dithiobis (2-nitrobenzoic acid) were then added to exosome fractions in a final volume of 300 μl, and the change in absorbance at 412 nm was monitored every 5 minutes for 30 minutes.

### Western blot analysis

For Western blot analysis, cells or exosomal preparations were lysed using lysis buffer (50 mM Tris (pH 7.5), 1% NP40, 0.25% DOC, 150 mM NaCl_2_, 1 mM PMSF, 10 μg/ml aprotinin/leupeptin/pepstatin, 20 mM NaF, 0.2 mM EGTA, 1 mM EDTA (pH 8.0), H_2_O). For protein concentrations, the BCA assay (Pierce, Rockford, IL) was used. Proteins from exosomes (20-40 μg) were separated using 12% Bis-Tris polyacrylamide gels, transferred onto polyvinylidene difluoride membranes (Millipore, Billerica, MA) and probed using the following antibodies: mouse monoclonal anti-LAMP1 (Abcam, Cambridge, MA), Anti-Survivin-2B, Anti-Survivin-∆Ex3 (Cell Signaling, Danvers, MA), and rabbit polyclonal anti-Survivin (Novus, Littleton, CO). Secondary antibodies (IR-Dye conjugated) were goat anti-rabbit and goat anti-mouse immunoglobulin (LICOR, Lincoln, NE). Immunoreactive bands were detected using the Odyssey Imaging System (LICOR, Lincoln, NE) and quantified using ImageQuant software.

### Immunohistochemistry

Pathological diagnosis of receptor status was performed by immunostaining for ER, PR, and Her2 as routine pathological procedures. Formalin fixed paraffin embedded tissues were cut into 4 micron sections. The detailed deparaffinization and immunohistochemistry protocols have been described previously [16]. Briefly, slides were stained using the commercially available anti-Survivin, Survivin-2B, and Survivin-∆Ex3 antibodies. Staining was performed manually in the following manner. Slides were deparaffinized through xylene and graded ethyl alcohol and then rinsed in water. Antigen retrieval was performed by boiling slides in antigen retrieval solution (Dako, Carpinteria, CA; pH 6.0) in a microwave oven at maximum power for 4 minutes and at half maximum power for 12 minutes, followed by a 30 minute cool-down and rinsing in wash buffer. Slides were then sequentially treated with the following reagents in a humidified chamber at room temperature: 10% normal goat serum for 30 minutes, anti-Survivin/-2B/-∆Ex3 antibody (1:100 dilution) overnight, and secondary antibody conjugated with Alexa Fluor 555 and 488 for 30 minutes for signal amplification. Nuclear staining was performed using DAPI containing mounting media for 5 minutes. Stained slides were analyzed for Survivin/-2B/-∆Ex3 antibody expressions.

### Image acquisition using laser-scanning confocal microscopy

Stained tumor tissues were imaged and analyzed with an Olympus FV 1000 laser scanning confocal imaging system mounted onto an Olympus 1×81 microscope (Olympus America Inc., PA). Microscopic data was acquired with a 20x objective lens. Staining intensities were confirmed by two investigators, working independently, including a pathologist.

### Statistical analysis

All comparisons for statistical significance were performed using a Student *t* test, with all p values representing two-tailed tests. Statistical software (Simple Interactive Statistical Analysis) was used. A value of P < 0.05 was considered statistically significant.

## Results

### Detection of survivin in cancer patient sera

**Figure 1:**
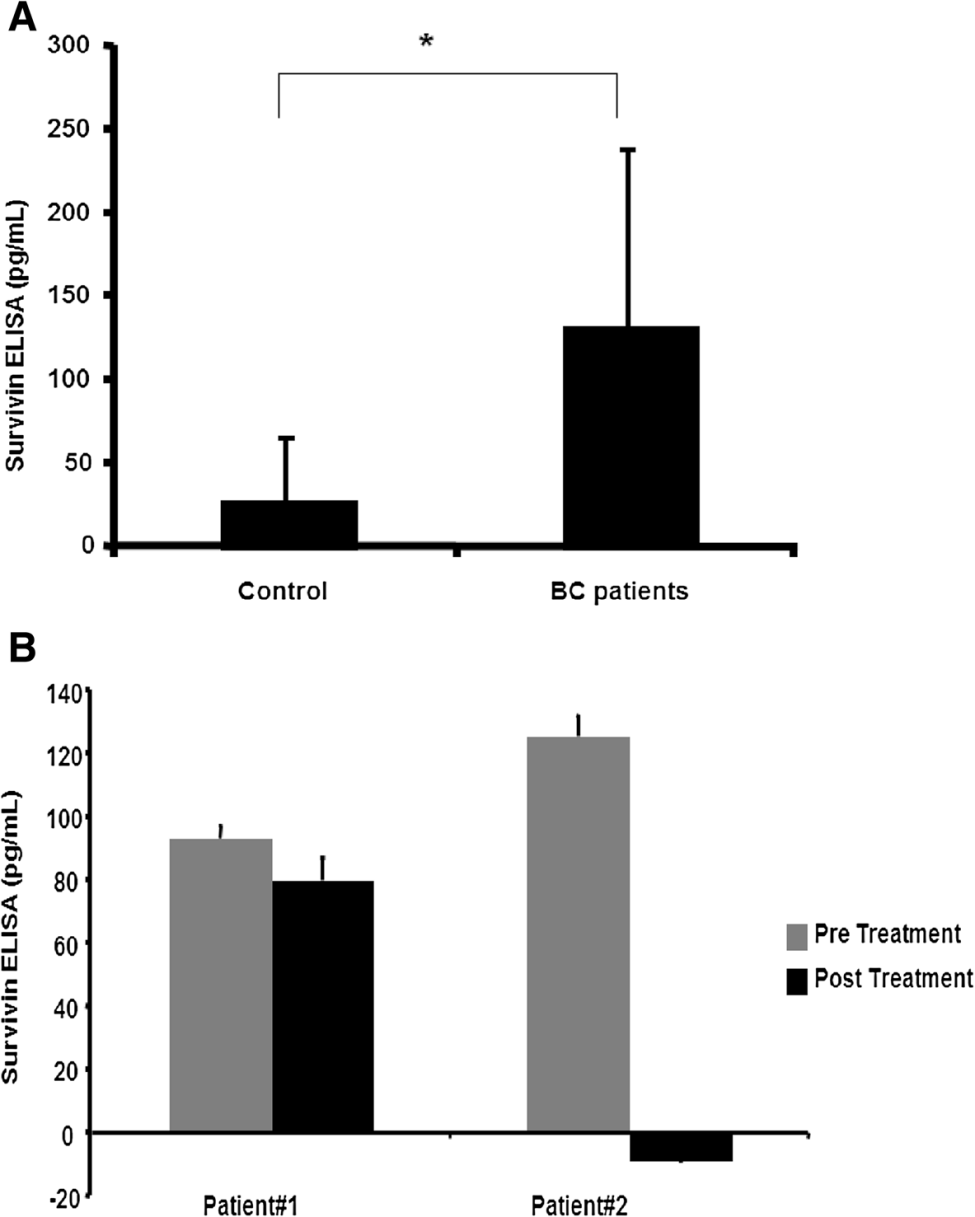
**Survivin quantification in patient sera. (A)** Survivin ELISA in control (with no recurrence after initial treatment) and breast cancer patients’ sera. Survivin level was compared with active breast cancer cases (n = 40) to controls (n = 10). Survivin values less than 50 pg/mL were considered normal while a Survivin level of 50-70 pg/mL was considered mild, 70-90 pg/mL moderate and >90 pg/mL as high. (*p < 0.05) **(B)** Treatment may affect patient sera Survivin levels. In two patients (patient#1), (patient#2) serum Survivin level are measured before and one year after treatment. (Error bar = Mean+/-SD). (BC, breast cancer).

Survivin protein levels as detected by ELISA were significantly (*p < 0.05) higher in cancer patient sera compared to controls (Figure 1A). Out of forty patient samples examined, 95% had moderate to high serum levels of Survivin. We also correlated Survivin levels from 2 patients sera obtained before and after treatment as shown in Figure 1B. Out of forty patient samples examined, 95% had moderate to high serum levels of Survivin. Serum Survivin was next correlated with tumor stage (Table 1). In Stage II/III and IV disease, moderate to high levels of Survivin were detected in 94% and 86% of total cases, respectively. No significant difference was detected in these stages. We next examined the serum level of Survivin in the context of patient hormone receptor status. Moderate to high serum levels of Survivin were detected in 86% of triple negative (ER-PR-Her2-) breast cancer, and 83% of patients with ER and PR positive, Her2 negative (ER + PR + Her2-) receptor status (Table 2).

**Table 1:** Relationship of serum survivin with clinical staging

**Survivin ELISA**	**Stage II/III**	**Stage IV**
Moderate to high (70 pg/ml)	17 (94%)	19 (86%)
Low normal to mild (<50 pg/ml)	1 (6%)	3 (14%)
Total	18	22

**Table 2:** Correlation of serum survivin levels with receptor status

**Survivin ELISA**	**ER + PR + Her2-**	**ER-PR-Her2-**	**ER + PR + Her2+**
Moderate to high (70 pg/ml)	19 (83%)	6 (86%)	1 (50%)
Low normal to mild (<50 pg/ml)	4 (17%)	1 (14%)	1 (50%)
Total	23	7	2

### Exosomes and exosomal survivin splice variants in breast cancer patient sera

**Figure 2:**
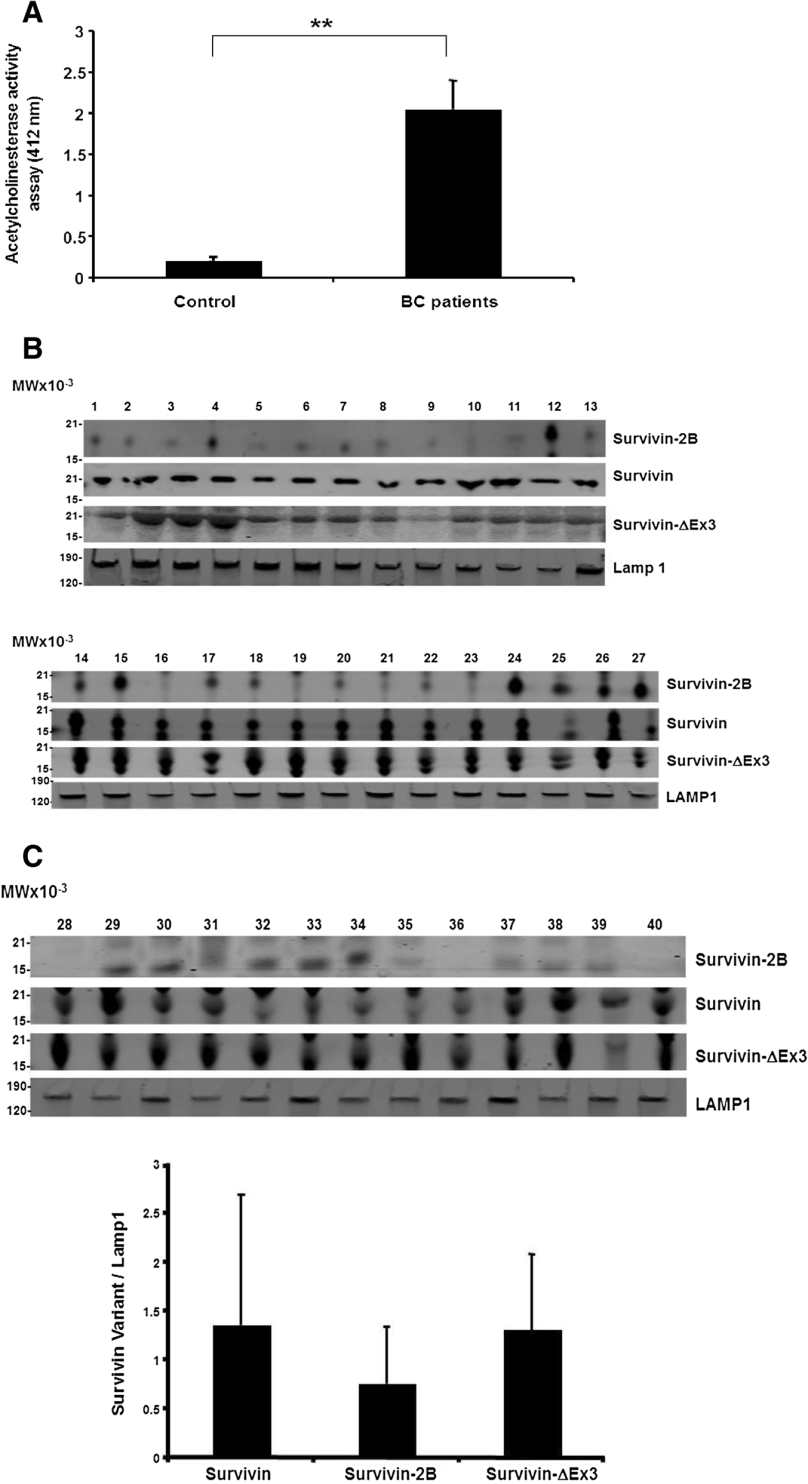
**Exosome level and exosomal contents from patient sera. (A)** Acetylcholinesterase Activity Assay: exosomes quantity was shown in the graph from sera of breast cancer patients (n = 40) with controls (n = 10) (**p < 0.01) (BC, breast cancer). **(B)** Western blot analysis of all exosomal Survivin, Survivin-2B, Survivin-∆Ex3, and LAMP1 from breast cancer patients sera (n = 40). **(C)** Densitometric analysis of Survivin, Survivin-2B, Survivin-∆Ex3, and LAMP1 expression was performed. A ratio of each Survivin variant to LAMP1 was calculated and depicted in the graph (error bar = mean +/- SD).

Exosomal analysis using the semiquantitative acetylcholinesterase activity assay showed that in sera taken from breast cancer patients (n = 40), cancer exosome amount is significantly (**p < 0.01) higher compared to exosome amounts in serum from control patients (n = 10) (Figure 2A).

Exosomes were also evaluated using Western blotting (Figure 2B) for the packaging of Survivin and its splice variant contents followed by densitometric analysis (Figure 2C) of all samples (N = 40). By densitometry, Survivin and Survivin-∆Ex3, using LAMP1 as the internal standard, were uniformly elevated in all patient exosomes. However, Survivin-2B, again standardized using LAMP1, was lower than Survivin and Survivin-∆Ex3 protein obtained from the same exosomes examined.

### Survivin-2B is differentially expressed in breast cancer tissues

**Figure 3:**
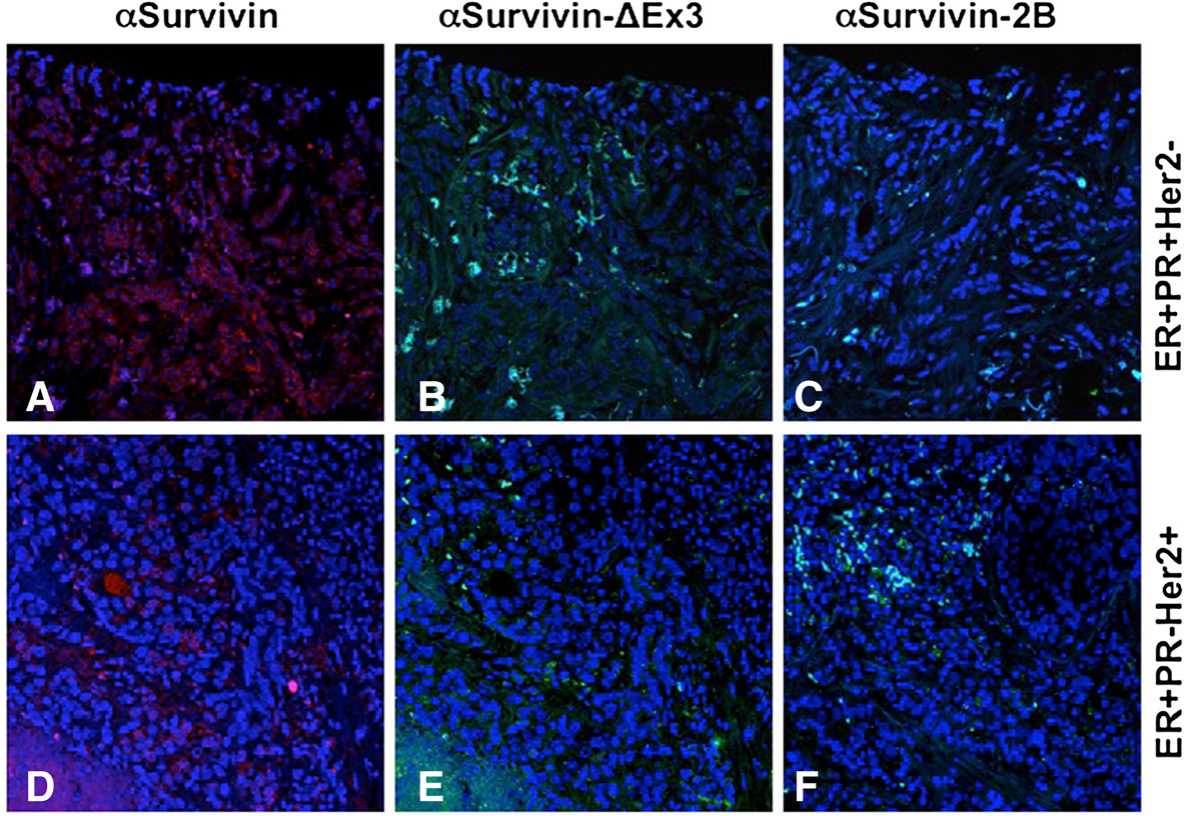
**Immunohistochemistry and confocal microscopy analysis of the survivin splice variants in breast cancer tissues: survivin (A, D), Survivin-∆Ex3 (B,E), and Survivin-2B (C,F).** The receptor status (ER + PR + Her2-) **(A-C)**, and (ER + PR-Her+) **(D-F)**. Original magnification is 20X. (ER, estrogen receptor; PR, progesterone receptor; Her2, human epidermal growth receptor type 2; α, antibody).

Survivin, Survivin-∆Ex3, and Survivin-2B expression and tumor grade is summarized in Table 3. Staining intensity was described as absent staining (0), low (+), moderate (++) and high (+++). In stage II/III disease, moderate (++) to high expression (+++) of Survivin was found in 89% and 94% in stage IV (metastasis) tumors. Similarly, moderate (++) to high (+++) Survivin-∆Ex3 expression was recorded in 100% of stage II/III, and 95% of stage IV disease. Conversely, Survivin-2B expression was low to absent in 75% of stage II/III diseases and absent in 100% of Stage IV diseases. For each type of breast tissue investigated, Survivin (Figure 3A & D) and Survivin-∆Ex3 (Figure 3B & E) were the predominant forms detected. Survivin-2B expression (Figure 3 C & F) was low in the tumor tissues examined.

**Table 3:** Relationship between expressions of survivin and its splice variants in tissues with clinical staging

**Characteristic**	**n**	**Survivin**	**Survivin-2B**	**Survivin-ΔEx3**
**Stage II/III**	7	+++ (62%)	+++ (0%)	+++ (50%)
		++ (27%)	++ (25%)	++ (50%)
		+ (11%)	+ (50%)	+ (0%)
		0 (0%)	0 (25%)	0 (0%)
**Stage IV**	9	+++ (75%)	+++ (0%)	+++ (70%)
		++ (19%)	++ (0%)	++ (25%)
		+ (6%)	+ (19%)	+ (5%)
		0 (0%)	0 (81%)	0 (0%)

**Figure 4:**
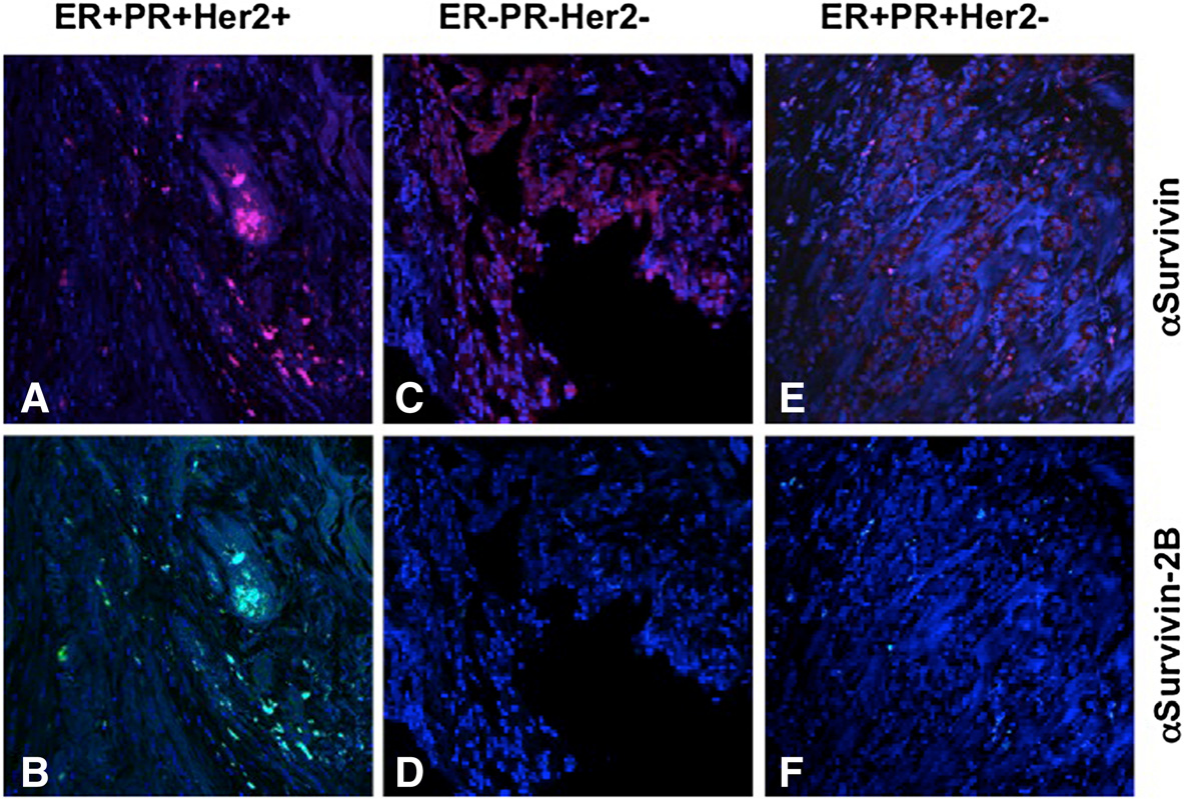
**Immuno-confocal microscopy of survivin splice variants in breast cancer tissues.** Triple positive (ER + PR + Her2+), triple negative (ER-PR-Her2-), and double positive, single negative (ER + PR + Her2-) breast cancer tissues were immune-stained with Survivin **(A, C, E)**, and Survivin-2B antibodies **(B, D, F)**. Original magnification is 20X.

In the early stages of the breast cancer tissues we investigated, comparable expression of both Survivin and Survivin-2B (Figure 4A &4B) was detected (Stage I). Although Survivin (Figure 4C) was strongly expressed in triple negative (ER-PR-Her2-) tumor tissues, Survivin-2B was absent (Figure 4D). In high-grade tumor tissue showing stronger Survivin expression (Figure 4E), low or no expression of Survivin-2B was recorded (Figure 4F).

**Figure 5:**
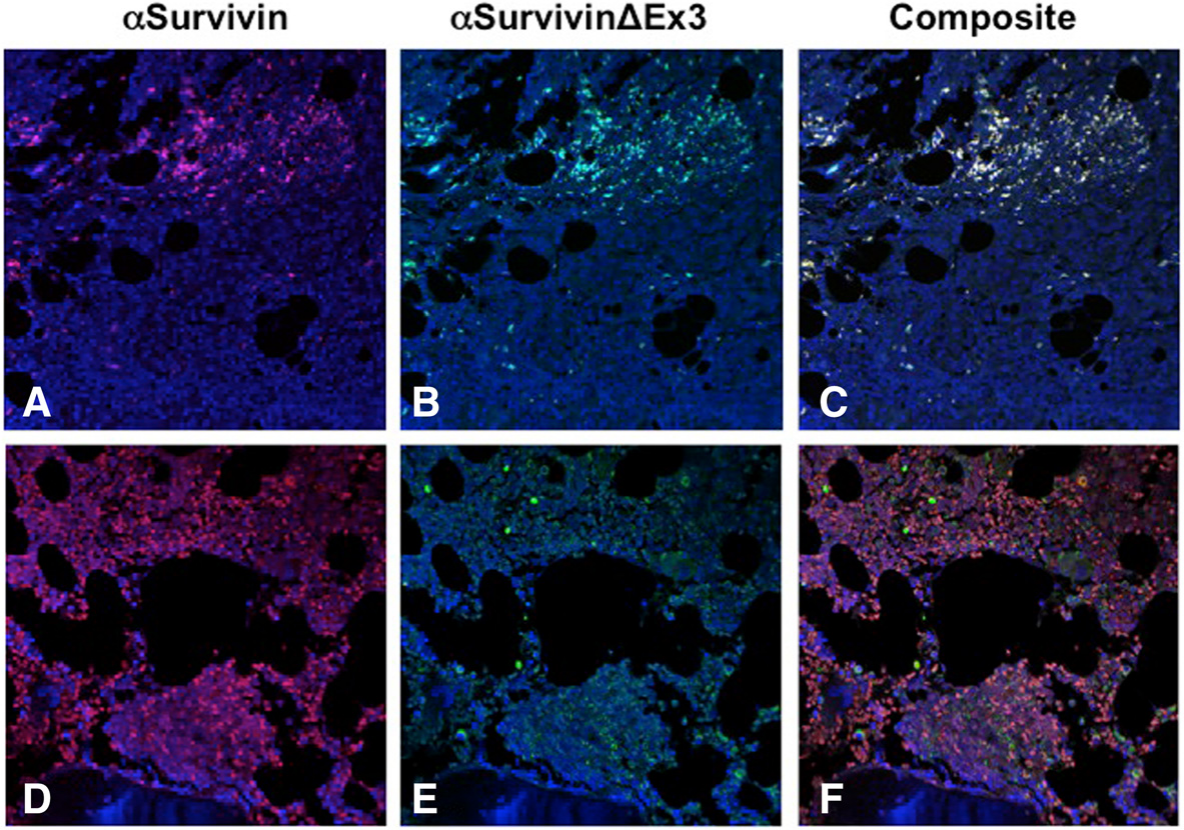
**Immuno-confocal microscopy of survivin splice variants in breast cancer tissues.** Survivin and Survivin-∆Ex3 expression are shown to colocalize in higher grades of breast tumor tissues **(A-C)** and distant (bone) metastasis **(D-E)**. Colocalization was shown when both stainings are superimposed as a yellow pattern **(C and F)**. Original magnification is 20X.

Lastly, Survivin and Survivin-∆Ex3 colocalize (Figure 5) in high-grade breast cancer tissues (Figure 5A-5C) and stage IV distant metastasis (Figure 5D-5F). Both proteins when colocalized are shown as a yellow staining pattern in the composites (Figure 5C and 5F). Although Survivin and Survivin-∆Ex3 proteins were predominantly expressed in cancer tissues, and distant metastasis, they were expressed in different grades of the tumor and expression pattern was similar in breast cancer tissues with different receptor status. However, an inverse correlation of Survivin-2B with tumor grade was recorded in these cancer tissues. A strong correlation with Her2-negativity with low or no Survivin-2B expression was also recorded. Minimal expression of Survivin-2B was found in normal adjacent tissues with no expression of Survivin or Survivin-∆Ex3 (**data not shown**).

## Discussion

Survivin has been recognized as an important molecular marker and target in a variety of cancer prognoses and therapeutics [17-19]. We have recently reported exosomal Survivin in the conditioned media (CM) from a number of cancer cell lines and in serum acquired from patients with prostate cancer [13]. This however, is the first report of Survivin and its splice variants Survivin-∆Ex3 and Survivin-2B in exosomes purified from breast cancer serum samples. In addition, the evaluation of this important family of apoptosis regulators in the treatment and understanding of breast cancer is also quite novel. Interaction of Survivin with its splice variants and the subcellular location where these interactions take place are crucial for validating Survivin as a target for cancer therapeutics, prognosis and probable prediction. Development of new model systems may be desired to unravel the mechanism for the functions of Survivin and its variants in tumorigenesis. In this work we have not yet fully identified the subcellular location of these Survivin splice variants, only the exosomal pool.

Currently, there is no strong evidence suggesting that Survivin splice variants are involved in tumorigenesis [20]. In this study, we evaluate the localization of Survivin and its splice variants in exosomes from breast cancer patients. We show that Survivin can coexist and possibly colocalize with its alternatively spliced protein products in the tumor tissues as well as in exosomes. This change in localization suggests the possibility of functionally distinct Survivin complexes that may arise within tumor cells that aberrantly express Survivin proteins at high levels. Importantly, the expression patterns observed in exosomes precisely mimic the patterns observed in tumor tissue. Currently there is rigorous research ongoing in the field of tumor biomarker discovery with exosomes playing an important role there [21,22]. Exosomal Survivin and Survivin splice variant measurement could serve as reliable biomarkers for the purpose of early diagnosis.

Although our current study is limited to stage II-IV patient populations, it may be of great interest to explore the diagnostic value of this novel tumor marker in stage I invasive breast cancer and ductal carcinoma in situ in the future. The exosomal packaging may suggest the possibility of functionally distinct Survivin and Survivin splice variants within tumor cells that aberrantly express high levels of these Survivin proteins. We suggest that targeting specific Survivin isoforms, rather than Survivin alone, may selectively and effectively destroy tumor cells. Recently, Caldas et al., reported that survivin-∆Ex3 and survivin-2B can heterodimerize with survivin DNA and alter its subcellular localization [9]. Islam et al., reported that the expression of survivin-2B is predominant in some neuroblastomas with good prognosis, but it is expressed at low levels in most malignant tissues [23]. In our study, we found that Survivin-2B expression was inversely related to tumor grade in breast cancer patient tissues or exosomes compared to Survivin and Survivin-∆Ex3.

Early stage breast cancer showed expression of Survivin-2B, but there was low or no expression detected in the later stages of the tumors. In patients whose Survivin-2B expression responded well to treatment, we are following up to correlate the role of Survivin-2B expression with good prognosis, as Survivin-2B seems to act as a natural antagonist [23,24] against the function of Survivin and/or Survivin-∆Ex3. If successful, Survivin-2B could be confirmed as a good prognostic marker in this disease. We will need to follow this up with a larger patient population.

In late stage breast cancer (Stage IV), with visceral organ or bone metastasis, expression of Survivin-2B in both tumors as well as in exosomes was low to absent. This may indicate an unfavorable role of Survivin-2B in breast cancer development and thus therapeutic efficiency may be enhanced by increasing Survivin-2B expression. Differential endogenous expression of Survivin, Survivin-∆Ex3, and Survivin-2B is reported in different cancers including breast cancers [6,9,25-27]. Although Ryan et al., reported that levels of both survivin-2B and survivin-∆Ex3 but not survivin were significantly higher in nodal metastases than primary carcinomas [6], we found the opposite in our tissue and exosomes with regard to Survivin variant proteins. Survivin and Survivin-∆Ex3 were the predominant forms expressed in almost all of the samples examined. This difference was particularly marked for Survivin-2B and may relate to the fact that by the advent of commercially available antibodies, we were able to identify different isoforms precisely in both exosomes and cancer tissues in this study. Therefore, it is desirable to detect each of the Survivin splice variant proteins and mRNA to determine breast cancer prognosis.

## Conclusion

In conclusion, exosomal Survivin and the Survivin splice variant expression patterns mimic that found in tumor tissue and thus may prove promise as tumor markers in the early detection of breast cancer. Also, we found that there is an inverse relationship between the staging of the breast cancer and expression of Survivin-2B, i.e., in the early stage when Survivin-2B is available patients are likely to have a favorable treatment outcome. In the later stages of the disease, constant expression of Survivin and Survivin-∆Ex3 throughout the disease stages may indicate an aggressive phenotype. Therefore, from a cancer prevention and therapeutics point of view, Survivin-2B, which is a proapoptotic protein [6], may act as a natural antagonist against the function of Survivin and/or Survivin-∆Ex3 leading to novel approaches for cancer prevention and/or therapeutics through differential modulation of the expression of Survivin and/or its variants. Although data from several reports suggest a potential role of Survivin-2B in countering the function of Survivin and/or Survivin-∆Ex3 in tumor development, much more needs to be done before deriving a definitive conclusion. Further study is underway in our lab to confirm the role of Survivin-2B as a breast cancer prognostic marker in a large cohort of breast cancer patients.

## Abbreviations

IAP: Inhibitor of apoptosis; BIR: Baculovirus IAP repeat; RT-PCR: Reverse transcription-polymerase chain reaction; CM: Conditioned medium; ER: Estrogen receptor; PR: Progesterone receptor; HER2: Human Epidermal Growth Factor Receptor 2, ELISA, enzyme-linked immunosorbent assay.

## Competing interests

The authors declare that they have no competing interests.

## Authors’ contributions

SK carried out the immunoassays, Western blots and drafted the manuscript. HFB aided SK with the Westerns. DT carried out the enzymatic assays. MP as our pathologist analyzed with SK the histologies. SM participated in the design of the study and aided in the collection of the patient samples. NRW with YY conceived of the study, and participated in its design and coordination and helped to draft the manuscript. All authors read and approved the final manuscript.

## Pre-publication history

The pre-publication history for this paper can be accessed here:

http://www.biomedcentral.com/1471-2407/14/176/prepub
